# Proteome Analyses of *Staphylococcus aureus* Biofilm at Elevated Levels of NaCl

**DOI:** 10.4172/2327-5073.1000219

**Published:** 2015-09-22

**Authors:** Nazrul Islam, Julia M Ross, Mark R Marten

**Affiliations:** Department of Chemical and Biochemical Engineering, University of Maryland Baltimore County (UMBC), 1000 Hilltop Circle, Baltimore, MD 21250, USA

**Keywords:** Biofilm, NaCl, *Staphylococcus aureus*, Elevated, Proteome

## Abstract

Our studies demonstrate that sodium chloride (NaCl) induces changes in biofilm, mediated by increased production of polysaccharides intercellular adhesion (PIA). We identified 12 proteins that showed higher abundance in increased level of NaCl. This includes one important protein (IsaA) known to be associated with biofilm stability. In addition, we also found higher abundance of a cold shock protein, CspA, at higher NaCl. We have also identified several other proteins that are differentially expressed to the elevated levels of NaCl and mapped them in the regulatory pathways of PIA. The majority of proteins are involved with various aspects bacterial metabolic function. Our results demonstrated that NaCl influences gene regulatory networks controlling exopolysaccharide expression.

## Introduction

Biofilm, an extracellular polymeric matrix secreted by certain microorganisms, has been found in 65%–80% of bacterial infections, and is considered refractory to host defenses in antibiotic therapy [1]. Among the biofilm-forming bacteria, *Staphylococcus aureus* is responsible for health problems ranging from minor skin infections to such major diseases as bacteremia, endocarditis and osteomyelitis. Treatments of such infections became more challenging when several *S. aureus* strains showed resistance to multiple antibiotics (e.g., methicilin and vancomycin). Community associated methicillin-resistant *S. aureus* has recently been recognized as one of the major causes of serious illness causing high morbidity, mortality and heath care costs [2]. The treatment of community associated biofilm infections costs over $1 billion annually in the USA [3].

Community-acquired infections total over 2 million for *S. aureus* [4], and the situation is becoming more alarming because some of the community-acquired strains are highly virulent [5]. A part of the community acquired infections occur through recreational areas such as swimming pools and beaches where people are exposed to saline water. A high correlation between saline water exposure and *S. aureus* infection rates was also reported [6,7]. In addition, infection from *S. aureus* is widespread in the respiratory tract of cystic fibrosis (CF) patients. Bacterial infection of the respiratory tract is widespread among cystic fibrosis (CF) patients. Although incompletely understood, the local environment in the CF airway appears to favor biofilm formation leading to chronic persistent infection [8,9]. Over time, infection coupled with an aggressive host inflammatory response contributes to irreversible damage of CF airways. Due to its rapidly emerging antibiotic resistance, *S. aureus* infections are increasingly difficult to treat. Methicillin-resistant strains of *S. aureus* (MRSA) now account for approximately 50% of all *S. aureus* infections [10] and the first cases of vancomycin-resistant *S. aureus* (VRSA) in the United States were reported in 2002 [11]. In a recent study of the respiratory microbiology of CF patients in the U.S., both the incidence (21.7% in 1995, 33.2% in 2005) and prevalence (37% in 1995, 52.4% in 2005) of infection due to *S. aureus* increased significantly (in contrast to *Pseudomonas aeruginosa*, which showed a significant decline) [12]. Fifty to 80% of CF children and adolescents are chronically colonized or infected by *S. aureus* [13]. Taken together, these trends suggest an urgent need to better understand the pathogenesis of staphylococcal infections in CF airways.

Chronic bacterial infections of CF airways occur because alterations to the lung airway surface liquid (ASL) result in thick secretions that cannot be well-removed by the lung. The ASL provides a nutrient rich environment for bacterial growth, colonization and biofilm formation and is a hyperosmotic environment with higher than normal NaCl concentrations [8,9]. Evidence in the literature suggests that increasing NaCl concentrations results in increased biofilm formation [14]. The formation of physically stable *S. aureus* biofilms in CF airways results in increased antibiotic resistance and decreased likelihood of infection eradication.

Biofilm stability is a function of its extracellular matrix, where both polysaccharide and protein play critical roles [15,16]. The majority of the matrix (90%) is composed of polysaccharide intercellular adhesin (PIA) [17–21]. Based on structure, two types of PIA have been reported. PIA type I (typically>80%) is a unique linear β-1,6 glucosaminoglycan which is predominantly positively charged. PIA type II (typically<20%) is structurally similar to type I, but contains phosphate and ester-linked succinate, and thus carries a mild negative charge [20,21]. The linear structure of these PIAs facilitates electrostatic interaction between positively and negatively charged residues which ultimately contributes to biofilm stability [21]. In addition, surface proteins appear to play a critical role in contributing to biofilm stability. For example, nearly all *S. aureus* clinical isolates possess and express the genes necessary for PIA production (icaoperon, described below), yet many do not form biofilms [22,23]. This implies that surface proteins may act as additional biofilm stabilizers, possibly cooperating with PIA to mediate intercellular adhesion [24]. While previous studies have demonstrated the importance of PIA, or implied the necessity of critical surface proteins for biofilm stability, the impact of increased levels of NaCl on biofilm architecture and stability is unknown.

The broad objective of this research is to analyze *Staphylococcus aureus* biofilm PIA and surface proteins at increased levels of NaCl. In particular, we tested the hypothesis that NaCl induced changes in biofilm architecture mediated by increased production of PIA and other surface proteins. As *S. aureus* biofilm plays a major role in pathogenesis, a more comprehensive understanding of biofilm PIA and surface proteins in saline environments will provide more effective biomarker discovery that leads to development of antimicrobial therapeutics to meet the challenges of biofilm-related infections.

## Methods

### Bacterial strain

*S. aureus* Philips, a biofilm-forming bacterium, was used in this study. This strain was isolated from a patient diagnosed with osteomyelitis and was successfully used for previous studies [25–27]. Secondary cultures was generated by inoculating 1 ml of overnight culture into 50 ml of TSB and growing at 37°C with constant rotation in shake flasks for four hours. The culture was then treated with 0%, 1% and 2% of NaCl and grown at 37°C with constant rotation for 20 hrs before harvest.

The growth of the bacterial strains was monitored by measuring the absorbance of the broth at 600 nm on a spectrophotometer. The cells were then harvested and re-suspended in phosphate-buffered saline (D-PBS; 138 mM NaCl, 2.7 mM KCl, pH 7.4). Cell concentrations was be determined using a Coulter Multisizer.

### Measuring PIA

Shake flask cultures was generated by inoculating 10 μl glycerol stock into 50 ml of tryptic soya broth (TSB). Cells were grown at 37°C with constant rotation for 4 hours before addition of NaCl and then grown 20 hrs. Two ml of the culture was transferred to a microtube and centrifuged at 10,000 xg for 10 min at 4°C. The cell plates were washed twice with 1 ml of PBS buffer each time. Cells were then resuspended in 100 μl of 0.5 M EDTA, pH 8.0 and boiled in hot water for 10 min at 100°C. The sample was then centrifuged at 10,000 xg for 10 min at 4°C. The clear supernatant was transferred to a new microtube. Quantification of the crude PIA was performed by a colorimetric method as described elsewhere [26]. 50 μl of the crude PIA was be transferred to a microtube and mixed with 25 μl of 80% w/v Phenol solution (Sigma-Aldrich). Following that 1 ml of concentrated sulphuric acid was added. The solution was allowed to stand for 10 min at room temperature, and absorbance was read at 490 nm. The amount of PIA was normalized by dividing by the number of cells used for extraction.

### Protein extraction

Cells were washed with PBS containing 0.1% sodium azide and then with PBS without azide, followed by a brief wash with digestion buffer containing 10 mM Tris HCl, 1 mM EDTA, 5 mM MgCl_2_. Approximately 5 × 10^9^ bacterial cells were resuspended in 1 ml of digestion mixture containing 35% raffinose, protease inhibitor cocktail (1 tablet/ml of digestion buffer), lysostaphin (5 units/ml) and then incubated at 37°C for 30 min. Cell debris were removed by centrifugation at 8,000 xg for 20 minutes and the supernatant was collected. After digestion and centrifugation, the digest was kept at −20°C overnight and then centrifuged at 8,000 xg for 20 min; precipitated raffinose was discarded. After digestion and centrifugation, the protein solution was subjected to ultrafiltration using the Millipore ultrafiltration tube and centrifuged as per manufacturer’s instructions. Protein concentration in the solution was determined using 2 D Quant (GE) and the resulting solution will be stored at −80°C for 2-DE.

### Two dimensional gel electrophoresis

In preparation for 2-DE, 150 μg proteins was resolubilized by adding standard sample solubilization buffers containing urea (8M), thiourea (2M), ASB 14 (1%), DTT (1%), and Carrier ampholytes (0.08%). The resulting solution was diluted to the desired volume with destreak rehydration solutions. Rehydration of IPG strips with the sample was carried out in the immobiline dry strip re-swelling tray (GE Healthcare) according to the manufacturer’s instructions. IPG strips of pH 3–11 (NL 24 cm) were used. The rehydrated strips were subjected to isoelectric focusing (IEF), performed using IPGphor operated at 20°C in gradient mode (97 kV hr). After focusing, the strips were stored at −80°C for later use. Prior to the second dimension SDS-PAGE, IPG strips were equilibrated for 15 minutes in equilibration solution (15 ml) containing 50 mM Tris-HCl, pH 8.8, 6M urea, 30% w/v glycerol, 2% w/v SDS and traces of bromophenol blue with 100 mg/10 ml (w/v) of DTT.

A second equilibration was carried out for 15 minutes by adding iodoacetamide (250 mg/10 ml) instead of DTT in equilibration solution. Second dimension vertical SDSPAGE was performed using large format (26.8 × 20.5 cm) gels (12.5% T/2.6% C) according to the manufacturer’s instructions. Electrophoresis was carried out with an initial constant voltage of 10 mA/gel applied for 30 minutes followed by 20 mA/gel for overnight until the bromophenol band exits the gel. The gels were stained with colloidal coomassie brilliant blue (BioRad). Gels were scanned as 12-bit TIFF images using Biorad GS-800 densitometer and analyzed by Nonlinear Dynamics SameSpots (v.3.2). Spot volumes were normalized by the software to a reference gel. At least three gels (biological replicates) for each treatment was used for analyses.

### Protein identification

For mass spectrometric identification, gel spots were excised, destained, and digested with sequencing grade trypsin (Promega). Peptide samples were analyzed by Nano ESI-MS/MS using LTQ (Finnigan, Thermo, USA). Nano LC was performed at reversed phase conditions using an Ultimate 3000 (Dionex corporation, USA) C18 column with a flow rate of 1 μl/min-5 μl/min in 70%–90% acetontrile containing 0.1% formic acid. MS and MS/MS data was collected and interrogated using SEQUEST against the NCBI non-redundant protein database for *S. aureus* providing peptide tolerance of 1.4 amu. Searched results were filtered using three criteria: distinct peptides, Xcorr vs charge state (1.50, 2.00, 2.50, 3.00) and peptide probability (0.001). The confirmation of the protein identification was based on the Xcorr value of more than 50 and Sf score for individual peptide of more than 0.8.

### Protein localization and function

The prediction of protein localization sites in cells was determined by PSORT, a computer program which analyzes the input sequence by applying the stored rules for various sequence features of known protein sorting signals. The transmembrane protein domain was predicted by TMpred (http://www.ch.embnet.org/software). To analyse functional categories of the identified proteins, we submitted each protein to the KEGG database (http://www.genome.ad.jp/kegg/pathway.html) using BRITE hierarchy. KEGG BRITE is a collection of hierarchical classifications of proteins based on their biological function.

## Results and Discussion

Few studies have been conducted to evaluate staphylococcal biofilm proteome analyses as a function NaCl concentration. Our studies on *S. aureus* response to NaCl have demonstrated that NaCl induces higher PIA production.

### Polysaccharides intercellular adhesins and biofilm of *S. aureus*

**Figure 1:**
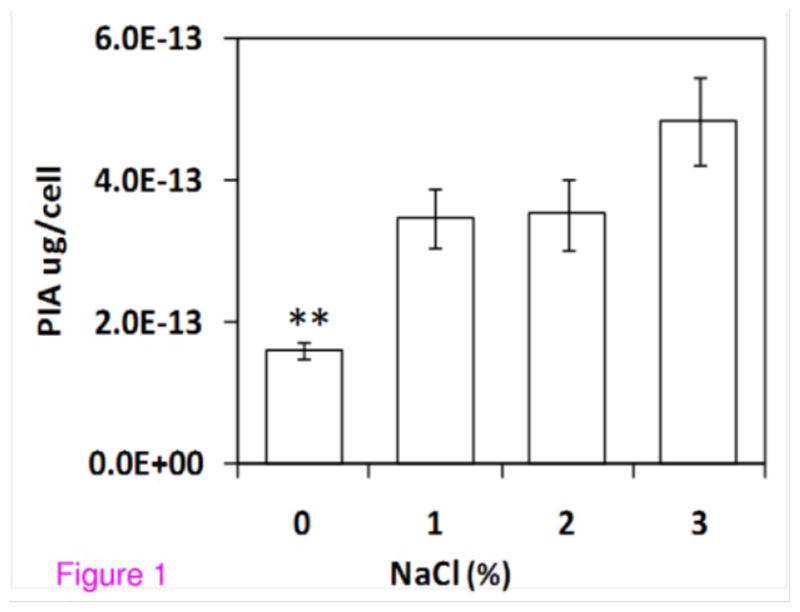
NaCl leads to increased PIA production. Plot of polysaccharide intercellular adhesion (PIA) per *S. aureus* cell for bacteria grown with increasing amounts of NaCl in the growth medium. Data for each treatment derived from three replicates. Bars represent standard error; **statistically significant difference compared to other bars (P<0.05).

We have developed an experimental protocol for isolation and quantification of polysaccharides intercellular adhesins of *S. aureus* by boiling cells with 0.5M EDTA, digesting the PIA with concentrated sulphuric acid and phenol, and then measuring absorbance at 490 nm. Although isolation of crude PIA by 0.5M EDTA is a routine procedure for PIA purification [28], to our knowledge it has not been reported for crude PIA quantification. We combined the EDTA extraction [28] with determination of sugars and their derivatives by colorimetry [29]. Using this procedure, we were able to reproducibly quantify PIA from *S. aureus*. As evident from the Figure 1, significantly higher amounts of PIA were observed with increased levels of NaCl in the growth media.

### Proteomics of *S. aureus*

**Figure 2:**
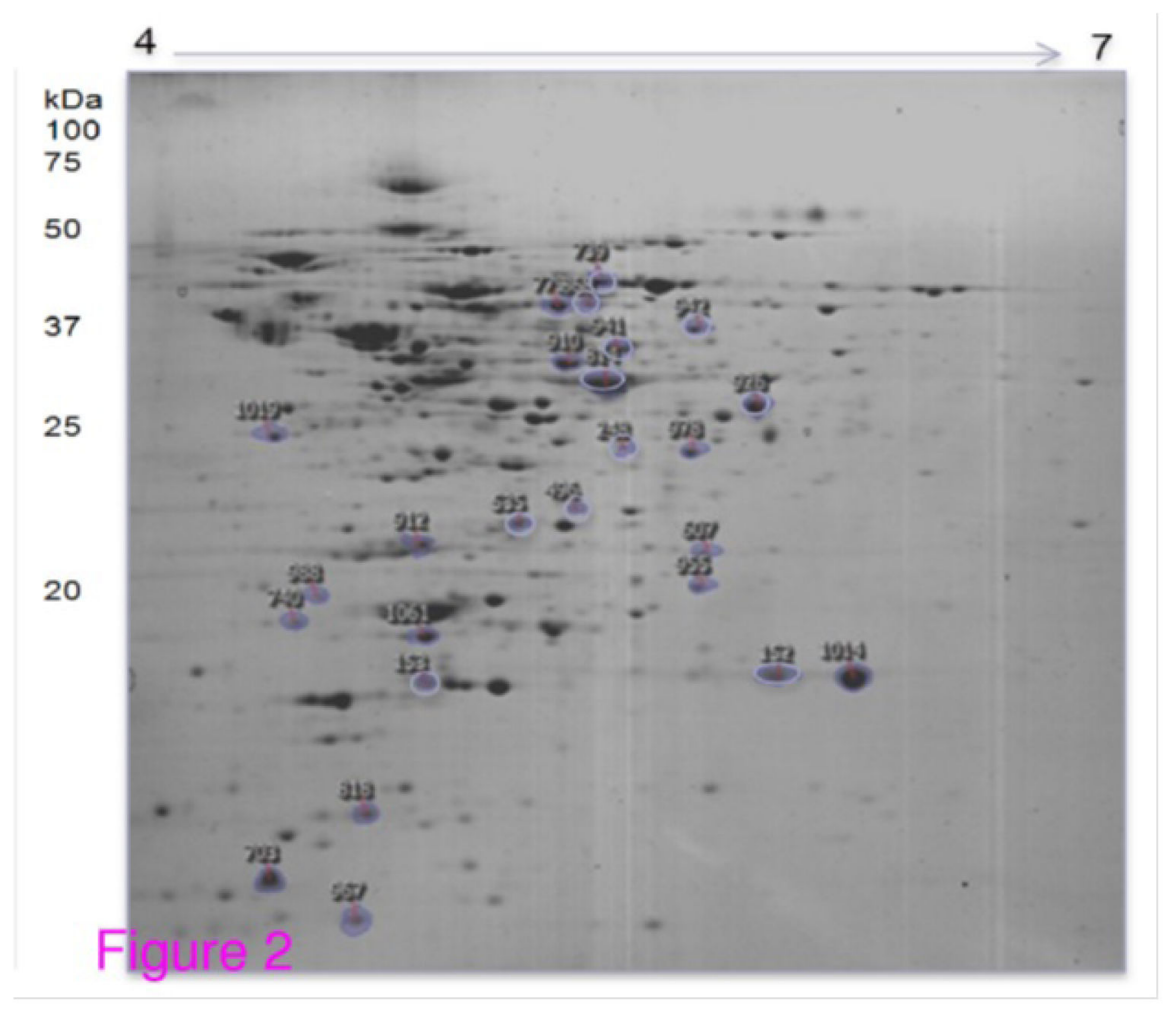
Typical two dimensional gel (pI and MW) of membrane associated proteins from *S. aureus* grown in increased NaCl concentration. Spot identifications are shown in Table 1.

In proteomic analysis, we grew *S. aureus* in shake flasks, with NaCl concentrations of 0%, 1%, 2%, 3% and 4%. In each case, cells were allowed to grow for 4 h before addition of NaCl, and then harvested after an additional 20 h growth. Membrane proteins were isolated as we’ve described previously [30] and these were then subjected to proteomic analysis via two dimensional gel electrophoresis. A representative gel is shown in Figure 2. We then used image analysis software to compare gels from the four different NaCl concentrations and were able to identify protein spots which showed continually increasing or continually decreasing abundance, with increasing NaCl concentration.

To analyze the effect of NaCl on protein expression, we considered proteins that showed progressively higher or lower abundance across the treatments. We identified 12 proteins that showed higher abundance in increased level of NaCl. This includes one important protein (IsaA) known to be associated with biofilm stability (Table 1). In addition, we also found higher abundance of a cold shock protein, CspA, at higher NaCl. Although this protein is implicated in the cells response to temperature, we believe it might play acrucial role in stabilizing biofilm through the network of PIA and surface proteins.

### PIA and surface protein biosynthesis and regulation

**Figure 3:**
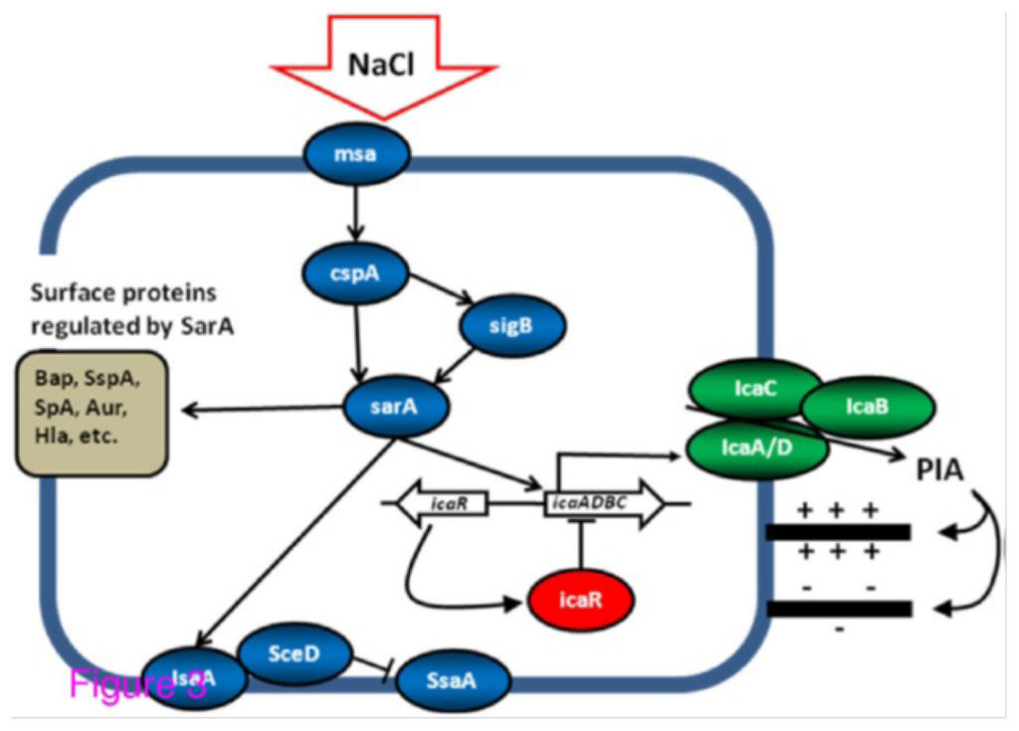
Network of genes, proteins and their regulatory relationships related to PIA production in *S. aureus* and relevant in this proposal. Details described in the text. Lines ending in arrows (â ) indicate activation or induction while those ending with lines (⊥) indicate deactivation or repression. Biofilm-associated protein, Bap [53]; a-toxin, Hla [54]; protein A (SpA) [55]; aureolysin (Aur) and serine protease (SspA) [56].

PIA production surface protein expression is controlled by a sophisticated regulatory network, with Figure 1 showing a number of the genes, proteins and regulatory relationships relevant to work proposed here. While many regulatory details are not completely understood, a number of the genes involved have been identified. A comprehensive network of PIA production based on others findings and two proteins (IsaA and CspA) identified in this investigation is shown in Figure 3.

PIA biosynthesis is mediated by ica operon-encoded enzymes [21,31]. The icaA, D and C gene products are involved in translocation of the growing polysaccharide to the cell surface [32], while IcaB is responsible for deacetylation of the PIA I molecule (providing its positive charge) which is essential for biofilm formation [33]. In contrast, the icaR gene, located upstream of the icaADBC operon, encodes a transcriptional repressor which plays a central role in the environmental regulation of the ica operon [34]. For example, exposure to NaCl activates the ica operon in an icaR-dependant manner [34–36].

Several global regulators are involved in PIA expression. Staphylococcal accessory regulator, sarA, controls expression of over 120 proteins [37], including a diverse range of virulence determinants [38], and is required for ica-operon transcription and PIA production [39,40]. SarA binds directly to the ica-operon promoter and positively regulates expression in an icaR-independent manner. SarA is likely to impact biofilm formation through other mechanisms as well. For example, expression of immunodominant staphylococcal antigen (IsaA) is regulated by SarA [41,42]. IsaA is involved in bacterial cell separation through a preferential interaction with peptidoglycan chains [41,43,44] and is known to ionically bind to the *S. aureus* wall [41]. Regulatory studies have shown IsaA and SceD, are mutually compensatory and that sceD is upregulated in the presence of NaCl [41]. Further, inactivation of isaA results in up-regulation of Staphylococcal Secretory Antigen A (ssaA), whose gene product has been shown to possess peptidoglycan hydrolase activity, and is thought to be important in biofilm formation. Finally, of the many proteins regulated by sarA, a number are known surface-related adhesive proteins [45]. It is possible that some of these play a role as biofilm stabilizers, interacting with PIA or facilitating intercellular interaction in as yet unknown ways.

The global stress response regulator, SigB, also plays a major role in biofilm development. While in *S. aureus* SigB does not directly interact with the ica operon [40] there is evidence of its interaction with sarA [36], implying an indirect regulatory roll. Relevant to work proposed here, sigB is activated by increased NaCl concentration [46]. Also relevant here, SigB activity leads to increased biofilm stability in S. epidermis [47] and it has been suggested [24] that SigB may play a role in *S. aureus* biofilm stability.

Another protein which may indirectly regulate ica-operon expression is the cold shock protein CspA. Cold shock proteins serve as nucleic acid chaperones, binding RNA and DNA, and thus likely facilitate the control of processes such as transcription and translation [48]. In *S. aureus*, CspA interacts with sigB, and appears to enhance its transcription [49]. Similar behavior has been observed in E. coli, where CspA regulates expression of at least 14 different genes [50]. CspA also appears to interact with sarA. In a screen for accessory elements that modulate expression of sarA, the putative membrane protein, msa, was identified and hypothesized to act as a sensor for external signal, increasing sarA expression through CspA. Relevant here, CspA has also been shown to be upregulated in the presence of increased NaCl concentration in gram positive, L. monocytogenes. [51].

Finally, a recent study implies additional, as yet unidentified, PIA regulatory proteins are likely to exist [52]. An insertional mutant library was created in a biofilm forming strain of *S. aureus*, and screened for strains with attenuated biofilm formation. The majority of mutations found outside the icaADBC locus were also associated with reduced PIA production. This clearly implies there are additional regulators of PIA production which remain to be described [24].

## Conclusions

Our preliminary studies demonstrate that NaCl induces changes in biofilm architecture mediated by increased production of PIA. We identified 12 proteins that showed higher abundance in increased level of NaCl. This includes one important protein (IsaA) known to be associated with biofilm stability. In addition, we also found higher abundance of a cold shock protein, CspA, at higher NaCl. Although this protein is implicated in the cells response to temperature, we believe it might play a crucial role in stabilizing biofilm through the network of PIA and surface proteins. We also mapped the differentially expressed protein in the regulatory pathways of PIA. The majority of proteins are involved with various aspects bacterial metabolic function. Our results demonstrated that NaCl influences gene regulatory networks controlling exopolysaccharide expression. The association of genes we identified in this study can be used to test the direct evidence in support of hypothesis that NaCl influences gene regulatory networks controlling polysaccharide expression, thereby altering biofilm architecture and increasing biofilm stability.

## Figures and Tables

**Table 1:** Proteins showing consistent increase, or decrease, in abundance with increasing Nil concentration. Gels similar to those shown in the Figure 3 were subjected to image analysis to identify differentially expressed proteins. These spots were then cut from the gels and proteins were identified via tandem mass spectrometry (MS). Cross correlation (XC) score is a metric associated with the quality of identification, with a score >20 indicating a positive identification; pI/MW is isoelectric point and molecular weight; Cov (%) is the fraction of protein sequence coverage identified via MS; Acce.No. is accession number for the protein.

Spot ID	Protein name/Gene name	Functional category	XC score	pl/MW	Cov (%)	Acce. No.
**Proteins with increased abundance**						
607	Immuno dominant antigen A/IsaA	Catalysis of the hydrolysis of any glycosyl bond	60	5.9/24	26	NP_373093
703	Major cold shock protein/cspA	Transcription	50	4.4/7	40	NP_371926
740	Hypothetical protein/SAV1875	Putative intracellular protease/amidase	100	4.4/19	60	NP_372399
779	Aldehyde dehydrogenase/SAV2122	Energy production and conversion	318	4.9/52	57	NP_372646
818	General stress protein 20U/dps	DNA-binding ferritin like protein	128	4.4/17	72	NP_372663
910	Phosphoglycerate kinase/pgk	Carbohydrate transport and metabolism	304	5.0/43	60	NP_371297
912	ABC transporter ATP-binding protein/SAV0842	Post-translational modification, protein turnover	216	4.7/28	76	NP_371366
955	3-oxoacyl-(acyl-carrier protein) reductase/fabG	Fatty acid biosynthesis	220	5.5/26	69	NP_371755
967	Hypothetical protein/SAV1067	Nucleotide transport and metabolism	120	4.6/10	98	NP_371591
1014	Universal stress protein family/SAV1710	Cellular processes and signalling	316	5.5/18	90	NP_372234
1019	Hypothetical protein/SAV2581	Predicted hydrolases or acetyltranferases	300	4.6/31	67	NP_373105
1061	Hypothetical protein/SAV1015	Information storgae and processing	188	4.7/19	65	NP_371539
**Proteins with reduced abundance**						
152	Universal stress protein(USP)/SAV1710	Cellular processes and signalling	80	5.5/19	60	NP_372234
153	Alkaline shock protein/asp23	Alkaline pH tolerance	134	4.9/19	56	NP_372706
248	Cysteine synthase/cysK	Aminoacid and transport and metabolism	138	5.2/33	49	NP_371037
535	Ribosomal subunit interface protein/RaiA	A stress response protein	100	5.0/22	41	NP_371276
739	Catalase/katA	ubiquitous enzyme protect cells from toxic effects	164	5.2/58	35	NP_646038
814	Ornithine-oxo acid transaminase/rocD	Aminoacid and transport and metabolism	298	5.1/43	58	NP_371481
868	Aldehyde dehydrogenase/SAV1875	Energy production and conversion	216	4.9/52	52	NP_372646
926	Alcohol dehydrogenase/adh1	Catalysis of rection related to alcohol to produce energy	320	5.2/36	65	NP_645385
941	2-amino-3-keto butyrate coenzyme A ligase/MW0505	Glycine, serine and threonine metabolism	198	5.1/43	46	NP_645322
942	Coenzyme A disulfide reductase/cdr	Catalyzes specifically the NADPH-dependent reduction	138	5.2/49	38	O52582
